# Supplementary material for: Quality risk management for microbial control in membrane-based water for injection production using fuzzy-failure mode and effects analysis
Source: PeerJ Comput Sci. 2024 Dec 23;10:e2565. doi: 10.7717/peerj-cs.2565 (PMC11784823; doi:10.7717/peerj-cs.2565)
Supplement: Supplemental Information 3 [file peerj-cs-10-2565-s003.docx]

Appendix 1 Fuzzy decision rules based on expert knowledge

| IF antecedent THE consequent | | | | | | | | | |
| --- | --- | --- | --- | --- | --- | --- | --- | --- | --- |
| Rule no. | Antecedent | | | Consequent | Rule no. | Antecedent | | | Consequent |
|  | Occurance | Severity | Detection | Rsik |  | Occurance | Severity | Detection | Rsik |
| 1 | Remote | Almost none | Certain | None | 66 | Moderate | High | Certain | Low |
| 2 | Remote | Almost none | High | None | 67 | Moderate | High | High | High low |
| 3 | Remote | Almost none | Medium | Very low | 68 | Moderate | High | Medium | Low medium |
| 4 | Remote | Almost none | Low | Low | 69 | Moderate | High | Low | High medium |
| 5 | Remote | Almost none | Very low | Low | 70 | Moderate | High | Very low | Low high |
| 6 | Remote | Low | Certain | Very low | 71 | Moderate | Very high | Certain | Low high |
| 7 | Remote | Low | High | Low | 72 | Moderate | Very high | High | Medium |
| 8 | Remote | Low | Medium | Low | 73 | Moderate | Very high | Medium | High medium |
| 9 | Remote | Low | Low | High low | 74 | Moderate | Very high | Low | Low high |
| 10 | Remote | Low | Very low | Low medium | 75 | Moderate | Very high | Very low | High |
| 11 | Remote | Medium | Certain | Very low | 76 | High | Almost none | Certain | None |
| 12 | Remote | Medium | High | Low | 77 | High | Almost none | High | Very low |
| 13 | Remote | Medium | Medium | Low | 78 | High | Almost none | Medium | Low |
| 14 | Remote | Medium | Low | High low | 79 | High | Almost none | Low | Low |
| 15 | Remote | Medium | Very low | High low | 80 | High | Almost none | Very low | High low |
| 16 | Remote | High | Certain | Low | 81 | High | Low | Certain | Very low |
| 17 | Remote | High | High | High low | 82 | High | Low | High | Low |
| 18 | Remote | High | Medium | Low medium | 83 | High | Low | Medium | High low |
| 19 | Remote | High | Low | Medium | 84 | High | Low | Low | Low medium |
| 20 | Remote | High | Very low | High medium | 85 | High | Low | Very low | Medium |
| 21 | Remote | Very high | Certain | High low | 86 | High | Medium | Certain | Low |
| 22 | Remote | Very high | High | Low medium | 87 | High | Medium | High | High low |
| 23 | Remote | Very high | Medium | Medium | 88 | High | Medium | Medium | Low medium |
| 24 | Remote | Very high | Low | High medium | 89 | High | Medium | Low | Medium |
| 25 | Remote | Very high | Very low | High | 90 | High | Medium | Very low | High medium |
| 26 | Low | Almost none | Certain | None | 91 | High | High | Certain | Low medium |
| 27 | Low | Almost none | High | None | 92 | High | High | High | Medium |
| 28 | Low | Almost none | Medium | Very low | 93 | High | High | Medium | High medium |
| 29 | Low | Almost none | Low | Low | 94 | High | High | Low | Low high |
| 30 | Low | Almost none | Very low | Low | 95 | High | High | Very low | High |
| 31 | Low | Low | Certain | Very low | 96 | High | Very high | Certain | Medium |
| 32 | Low | Low | High | Low | 97 | High | Very high | High | High medium |
| 33 | Low | Low | Medium | High low | 98 | High | Very high | Medium | Low high |
| 34 | Low | Low | Low | Low medium | 99 | High | Very high | Low | High |
| 35 | Low | Low | Very low | Medium | 100 | High | Very high | Very low | Very high |
| 36 | Low | Medium | Certain | High low | 101 | Very high | Almost none | Certain | Very low |
| 37 | Low | Medium | High | Low medium | 102 | Very high | Almost none | High | Very low |
| 38 | Low | Medium | Medium | Medium | 103 | Very high | Almost none | Medium | Low |
| 39 | Low | Medium | Low | High medium | 104 | Very high | Almost none | Low | Low |
| 40 | Low | Medium | Very low | Low high | 105 | Very high | Almost none | Very low | High low |
| 41 | Low | High | Certain | Low medium | 106 | Very high | Low | Certain | Low |
| 42 | Low | High | High | Medium | 107 | Very high | Low | High | High low |
| 43 | Low | High | Medium | High medium | 108 | Very high | Low | Medium | High low |
| 44 | Low | High | Low | Low high | 109 | Very high | Low | Low | Low medium |
| 45 | Low | High | Very low | High | 110 | Very high | Low | Very low | Medium |
| 46 | Low | Very high | Certain | Low medium | 111 | Very high | Medium | Certain | High low |
| 47 | Low | Very high | High | Low medium | 112 | Very high | Medium | High | High low |
| 48 | Low | Very high | Medium | Medium | 113 | Very high | Medium | Medium | Low medium |
| 49 | Low | Very high | Low | High | 114 | Very high | Medium | Low | Medium |
| 50 | Low | Very high | Very low | High | 115 | Very high | Medium | Very low | High medium |
| 51 | Moderate | Almost none | Certain | Very low | 116 | Very high | High | Certain | Low medium |
| 52 | Moderate | Almost none | High | Very low | 117 | Very high | High | High | Medium |
| 53 | Moderate | Almost none | Medium | Low | 118 | Very high | High | Medium | High medium |
| 54 | Moderate | Almost none | Low | High low | 119 | Very high | High | Low | Low high |
| 55 | Moderate | Almost none | Very low | Low medium | 120 | Very high | High | Very low | High |
| 56 | Moderate | Low | Certain | Low | 121 | Very high | Very high | Certain | High medium |
| 57 | Moderate | Low | High | High low | 122 | Very high | Very high | High | Low high |
| 58 | Moderate | Low | Medium | Low medium | 123 | Very high | Very high | Medium | High |
| 59 | Moderate | Low | Low | Medium | 124 | Very high | Very high | Low | Very high |
| 60 | Moderate | Low | Very low | High medium | 125 | Very high | Very high | Very low | Very high |
| 61 | Moderate | Medium | Certain | Low medium |  |  |  |  |  |
| 62 | Moderate | Medium | High | Medium |  |  |  |  |  |
| 63 | Moderate | Medium | Medium | High medium |  |  |  |  |  |
| 64 | Moderate | Medium | Low | Low high |  |  |  |  |  |
| 65 | Moderate | Medium | Very low | High |  |  |  |  |  |
