# Supplementary material for: Quality risk management for microbial control in membrane-based water for injection production using fuzzy-failure mode and effects analysis
Source: PeerJ Comput Sci. 2024 Dec 23;10:e2565. doi: 10.7717/peerj-cs.2565 (PMC11784823; doi:10.7717/peerj-cs.2565)
Supplement: Supplemental Information 4 [file peerj-cs-10-2565-s004.docx]

Appendix 2 Prevention and control measures for microbial risks in membrane-based WFI systems

| Code | Failure mode | Preventive actions and suggestions |
| --- | --- | --- |
| FM1 | Raw water quality does not meet requirement, such as increased levels of particulates, bacteria, organic matter, minerals, etc. | 1. Regular inspection of raw water supply pipelines; 2. Provide online facility for adding sodium hypochlorite solution to raw water; 3. Routine sampling of the raw water should be carried out. |
| FM2 | Unable to filter raw water | 1. Regular maintenance of multi-media filter; 2. Provision of alarm on multi-media filter; 3. Validation and confirmation of sanitation methods and frequency; 4. Defined periodic media replacement. |
| FM3 | Unable to filter raw water | 1. Regular maintenance of activated carbon filter; 2. Provision of alarm on activated carbon filter; 3. Validation and confirmation of sanitation methods and frequency; 4. Defined periodic media replacement; 5. Strong recommendation to avoid using carbon filter (ISPE 2021). |
| FM4 | Low concentration of chemicals in the antiscalant dosing tank, unable to remove precipitable ions | 1. Alarm provision is provided in case of low chemical; 2. Operation of the Chemical dosing system shall be verified during commissioning; 3. Routine checkup of chemical dosing shall be performed regularly. |
| FM5 | Low/high dosing of SMBS/SBS | 1. Alarm provision is provided in case of low/high chemical; 2. Operation of the Chemical dosing system shall be verified during commissioning; 3. Routine checkup of chemical dosing shall be performed regularly. |
| FM6 | Water Pressure may not sufficient to feed RO | 1. Provision of high-pressure pump with VFD; 2. Installation of pressure gauge to monitor RO feed water pressure; 3. Operation of the high-pressure pump shall be verified during commissioning; 4. Routine checkup of chemical dosing shall be performed regularly. |
| FM7 | Leakage of the RO membrane | 1. RO system integrity testing; 2. Monitoring instruments (e.g., pressure gauges and thermometers) and interlocks, such as a temperature switch or sensor in RO feed pump line to inhibit pump operation at high feed water temperatures to avoid membrane damage; 3. Calibration of measuring instruments; 4. Monitoring of salt rejection and permeate conductivity; 5. Training of operators and establishment of clear operating procedures. |
| FM8 | Damage to RO membrane | 1. Monitoring instruments (e.g., pressure gauges and thermometers) and interlocks, such as a temperature switch or sensor in RO feed pump line to inhibit pump operation at high feed water temperatures to avoid membrane damage; 2. Calibration of monitoring instruments; 3. Monitoring of salt rejection and permeate conductivity; 4. Instrumentation to monitor and alarm for presence of chlorine and interlock, such as online ORP analyzer; 5. Robust control of chlorine removal in pretreatment; 6. Training of operators and establishment of clear operating procedures. |
| FM9 | Scaling/fouling of RO membrane | 1. Monitoring of salt rejection and permeate conductivity; 2. Monitoring of operating pressure, transmembrane pressure, permeate flow; 3. regular cleaning of membrane; 4. Installation of alarm to alert abnormal RO process parameters; 5. Increase the flow velocity paralleling membrane surface since rate of membrane fouling is closely related to the velocity (Bilad et al. 2014; Kruschitz & Nidetzky 2020; Pervov 2016). |
| FM10 | CEDI cannot provide electric field | 1. Regular check-up and maintenance of system operation; 2. Monitor and interlock dilute conductivity; 3. Recommended to install medium pressure UV light upstream the CEDI (ISPE 2022). |
| FM11 | Leakage of CEDI membrane | 1. Provision of conductivity sensor downstream of CEDI to monitor stack resistance and dilute conductivity; 2. Monitoring and maintaining appropriate operating pressure to prevent water hammer; 3. Regular inspection for leakage. |
| FM12 | Leakage of UF | 1. Integrity testing of membrane; 2. Monitoring instruments and interlocks, such as monitor flow rate, pressure drop and temperature; 3. Calibration of monitoring instruments. |
| FM13 | Damage to UF membrane | 1. Monitoring instruments (e.g., pressure gauges and thermometers) and interlocks, such as a temperature switch or sensor in UF feed pump line to inhibit pump operation at high feed water temperatures to avoid membrane damage; 2. Calibration of monitoring instruments; 3. Robust control of chlorine removal in pretreatment; 4. Define cleaning and sanitation method and frequency; 5. Training of operators and establishment of clear operating procedures. |
| FM14 | Fouling of UF | 1. Provision of a pump with required flow rate for UF backwashing and rapid flushing; 2. Monitoring of operating pressure and permeate flow; 3. Define cleaning and sanitation method and frequency. |
| FM15 | Unable remove microorganism in the air | 1. Periodic testing of seal of the vent filter to avoid leakage; 2. The material of vent filter is hydrophobic, high temperature resistant and oxidation resistant; 3. Provision medium pressure UV light in water supply pipeline. |
| FM16 | Insufficient sealing | Regular inspection for leaks. |
| FM17 | Unpolished pipeline | 1. Passivation of pipelines after welding; 2. Qualified personnel for polishing operations; 3. Provision of passivation test report. |
| FM18 | Insufficient pump power | Regular performance inspection and maintenance of pump. |
| FM19 | Inadequate welding leading to pipeline leakage | 1. Welding by qualified welders; 2. Validation report of welding quality, such as pressure testing. |
| FM20 | Materials with water solubility | 1. All metal contact parts (storage tank and distribution pipelines, valves, sampling and user valves) and instrument contact parts should be made of SS 316L stainless steel; 2. Electro-polishing and orbital welding for distribution system pipelines; 3. Use of sanitary or hygienic design for flanges, unions, or valves. |
| FM21 | Inability to sanitize the system; ineffective sanitation; infrequent sanitation. | 1. Routine monitoring of the sanitation system; 2. Define sanitation method and frequency; |
| FM22 | Critical equipment process parameters cannot be monitored. | Regular performance confirmation and maintenance. |
| FM23 | Water quality cannot be measured. | Regular performance confirmation and maintenance. |
| FM24 | Measuring instruments cannot be calibrated. | Regular performance confirmation and maintenance. |

**References**

Bilad MR, Arafat HA, and Vankelecom IFJ. 2014. Membrane technology in microalgae cultivation and harvesting: A review. *Biotechnology advances* 32:1283-1300. DOI: 10.1016/j.biotechadv.2014.07.008

ISPE. 2021. *ISPE D/A/CH Affiliate: Production of water for Injection without distillation handbook*. USA: International Society for Pharmaceutical Engineering.

ISPE. 2022. *Good practice guide: Membrance-based water for injection systems*. USA: International Society for Pharmaceutical Engineering.

Kruschitz A, and Nidetzky B. 2020. Downstream processing technologies in the biocatalytic production of oligosaccharides. *Biotechnology advances* 43:107568. DOI: 10.1016/j.biotechadv.2020.107568

Pervov A. 2016. The influence of hydrodynamic factors, membrane surface properties and channel geometries on membrane performance and fouling mechanisms. In: Andreev V, editor. 5th International Scientific Conference “Integration, Partnership and Innovation in Construction Science and Education”. Moscow, Russia: EDP Sciences. p 03006.
